# Ancestry specific associations of FTO gene variant and metabolic syndrome: A longitudinal ARIC study

**DOI:** 10.1097/MD.0000000000018820

**Published:** 2020-02-07

**Authors:** Dale S. Hardy, Jane T. Garvin, Tesfaye B. Mersha, Susan B. Racette

**Affiliations:** a Department of Internal Medicine, Morehouse School of Medicine, Atlanta Georgia; b School of Nursing, University of Saint Augustine for Health Sciences, Saint Augustine, Florida; c Department of Pediatrics, Cincinnati Children's Hospital Medical Center, University of Cincinnati, Cincinnati, Ohio; d Program in Physical Therapy and Department of Medicine, Washington University School of Medicine, St. Louis, Missouri.

**Keywords:** ancestry, blood pressure, BMI, FTO rs9939609, longitudinal, metabolic syndrome, physical activity, race

## Abstract

Cross-sectional studies indicate that the fat mass and obesity-associated (FTO) rs9939609 gene variant is associated with metabolic syndrome (MetS) primarily in European ancestry. However, the association is not fully elucidated in African Americans.

We hypothesized that rs9939609 (AT = moderate-risk carriers or AA = high-risk carriers compared to TT = low-risk carriers) is associated with MetS and its component risk factors over time; and that its association is ancestry-specific. A secondary hypothesis was that higher levels of physical activity can decrease the deleterious effect of rs9939609 at higher body mass index (BMI).

Atherosclerosis Risk in Communities study repeated measures data from 4 visits (1987–1998) were obtained from the database of Genotypes and Phenotypes for 10,358 participants (8170 Whites and 2188 African Americans) aged 45 to 64 years at baseline. Guidelines for elevated blood pressure by the American College of Cardiology and American Heart Association Task Force were updated within the MetS criteria. Risk ratios (RR) and 95% confidence intervals from generalized estimating equations assessed population-average risks.

MetS was present among 3479 (42.6%) Whites and 1098 (50.2%) African Americans at baseline, and 50.3% Whites and 57% African Americans over 11-years of follow-up. Among MetS component risk factors, high waist circumference was most prevalent among White AT (RR = 1.07; 1.06–1.09) and AA (RR = 1.12; 1.10–1.14) higher-risk carriers. High triglycerides were elevated among African American AA high-risk carriers (RR = 1.11; 1.02–1.21) compared to TT low-risk carriers. Over time, White AT-and AA higher-risk carriers had 1.07 and 1.08-fold increase (*P* < .0001) in MetS risk. Physical activity had independent protective effects on MetS among both races (*P* < .05). White AA high-risk carriers with normal BMI and low vs high physical activity had higher MetS risk (RR = 1.69; 1.25–2.30 and RR = 0.68;0.53–0.87, respectively). In rs9939609 × BMI× physical activity interaction, White A-allele high-risk carriers had lower MetS risk (RR = 0.68; 0.53–0.87). Among Whites, physical activity can lessen the effect of rs9939609 and high BMI on risk for MetS.

## 1 Introduction

Metabolic syndrome (MetS), an atherosclerotic disease precursor defined by the National Cholesterol Education Program Adult Treatment Panel III as the clustering of 3 or more cardiovascular metabolic risk factors,^[1]^ approximately doubles the risk of type 2 diabetes, heart disease, stroke, cardiovascular mortality, and all-cause mortality.^[2–4]^ MetS is characterized by the presence of at least 3 of 5 risk factors that include abnormal values for waist circumference, blood pressure, fasting blood glucose, HDL cholesterol, and triglycerides. These risk factors give an indication of overall health and functioning of a patient's metabolic and circulatory systems, informing the clinician of changes in health status over successive visits.^[1]^ Updated guidelines for blood pressure by the American College of Cardiology (ACC) and American Heart Association (AHA) Task Force^[5]^ have lowered the blood pressure cut-points to designate elevated systolic blood pressure as ≥120 mm Hg (previously ≥130 mm Hg) and elevated diastolic blood pressure as ≥80 mm Hg (previously ≥85 mm Hg). Should the National Cholesterol Education Program ATP III Guidelines adopt the lower blood pressure cut-points, the prevalence of MetS may increase.

Reports using the old blood pressure cut-point for MetS (≥130/85 mm Hg) show that approximately 34% of adults have MetS,^[2]^ with a higher prevalence in older adults and in those with low levels of physical activity.^[6]^ Physical activity of moderate-intensity, performed 120 to 150 minutes per week, can reduce the risk of developing MetS, type 2 diabetes, cardiovascular disease, and stroke.^[2]^ Furthermore, lower levels of physical activity over a 20-year period were associated with elevated metabolic risk factors contributing to MetS.^[7]^ MetS increases with age, correlates with dyslipidemia, and is most prevalent among those with less than high school education.^[2,8]^ Conflicting reports show the age-adjusted prevalence of MetS in 2009 to 2010 was similar among Whites and African Americans (21.8%),^[2]^ compared to a previous report that showed a disparity of 37.2% for Whites and 25.3% for African Americans.^[9]^

The fat mass and obesity-associated (FTO) rs9939609 gene variant has the highest minor allele frequency compared to other FTO variants. It is one of the most replicated and reported variant in the GWAS catalog (6 studies in multiple ancestries). In addition, it has comparable minor allele frequency (MAF) in our study population: African (MAF = 0.46), African American (MAF = 0.44), and European (MAF = 0.43) ancestry samples. Furthermore, rs9939609 is functionally important as shown in HaploReg, including its role in Pou5f1 transcriptional activity. FTO rs9939609 is the strongest genetic variant associated with high body mass index (BMI), physical inactivity, and other abnormal metabolic parameters in various populations.^[10–25]^ However, the relationship between rs9939609 and abnormal metabolic traits (i.e., elevated waist circumference, hip circumference, waist-to-hip ratio, blood pressure, and dyslipidemia) in some studies became non-significant after adjustment for BMI due to its mediating effects.^[17,22,25–29]^

In a meta-analysis, rs9939609 variant was associated with an 11% increased risk of MetS in Whites.^[30]^ A cross-sectional study found an interaction between rs9939609 and physical activity on adiposity in Whites and African Americans.^[24]^ In that study, each additional copy of the high-risk rs9939609 A-allele was associated with higher obesity-related anthropometric traits in White and African American men. Another study that followed Korean adults for 6 years did not find an interaction between rs9939609 and MetS. However, in that study, BMI × rs9939609 interaction showed that participants with BMI ≥29 kg/m^2^ had higher risk for MetS.^[31]^

Thus, there is evidence that rs9939609, elevated BMI, and physical inactivity are interrelated and associated with MetS in cross-sectional studies.^[11,12,15,19,21,24]^ However, these studies have been performed primarily on populations with European (White) ancestry, and associations with rs9939609 and MetS have not been fully elucidated in other racial ancestry populations, specifically African Americans. The aim of this current study was to investigate whether FTO rs9939609 variant is associated with increased risk for MetS and its component risk factors from baseline to 11 years of follow-up in European ancestry (Whites) and African Americans. We hypothesized that rs9939609 variant increases the prevalence of MetS and its component risk factors in Whites and African Americans over time and that the magnitude of its association is ancestry-specific. A secondary hypothesis was that higher levels of physical activity can decrease the deleterious effect of rs9939609 variant in participants with higher BMI.

## 2 Methods

### 2.1 Study population

Atherosclerosis Risk in Communities (ARIC) study data at baseline (1987–1989) and 3 follow-up visits (1990–1992, 1993–1995, and 1996–1998) were obtained from the database of Genotypes and Phenotypes (dbGaP).^[32]^ The ARIC study, sponsored by the National Heart, Lung, and Blood Institute, is a large-scale ongoing prospective cohort study being conducted in 4 U.S. communities: Jackson, MS; Forsyth County, NC; Minneapolis, MN; and Washington County, MD. ARIC was designed to investigate the etiology and natural history of atherosclerosis, as well as the causes of clinical atherosclerotic diseases and their sequelae. Participants included White and African American men and women who were 45 to 64 years old at baseline.^[33]^ Further design and sampling methods are explained elsewhere.^[34]^ All participants signed an informed consent prior to participation in ARIC data collection. The present study was approved by Morehouse School of Medicine Social and Behavioral Institutional Review Board, Atlanta, Georgia, USA.

### 2.2 Study variables

MetS was designated as the outcome variable, defined according to the modified National Cholesterol Education Program Adult Treatment Panel III criteria^[35]^ as clusters of ≥3 of the following cardiovascular disease risk factors: waist circumference >102 cm in men or >88 cm in women, systolic blood pressure ≥120 mm Hg or diastolic blood pressure ≥80 mm Hg or on high blood pressure medication, fasting blood glucose ≥100 mg/dl or diabetes diagnosis or on diabetes medication, HDL cholesterol <40 mg/dl in men or <50 mg/dl in women, and triglyceride level ≥150 mg/dl or on lipid controlling medication.^[2–4]^ BMI (continuous variable) was calculated as weight in kilograms divided by height in meters squared (kg/m^2^). Leisure-time sport physical activity (continuous variable) was determined from the validated Baecke self-administered questionnaire that gathered information on habitual physical activity.^[36]^ Leisure-time sports physical activity data from baseline visit 1 (1987–1989) were carried forward to visit 2, and physical activity data from visit 3 was duplicated and carried forward to visit 4 (1993–1995) because these data were collected only at visits 1 and 3. Education level (3 level categorical variable) was collected at visit 1 only. Age (continuous variable), gender, duration of study time (a numerical sequential indicator for each visit (1 to 4)), and the first 3 genetic principal components (continuous variable - used to correct for population genetic admixture) were included as additional covariates. FTO rs9939609 variant was presented as the main predictor variable whose values were equal to the number of copies of the high-risk A-allele under the additive model (i.e., 0 = TT low-risk, 1 = AT moderate-risk, and 2 = AA high-risk carriers). BMI and leisure-time sport physical activity were defined as the interaction variables. All analyses were stratified by race, which was self-reported as White or African American. In other analyses that involved interaction, race, and gender were the stratifying variables.

### 2.3 DNA extraction and genotyping

Based on dbSNP (single nucleotide variant database) data, the FTO rs9939609 variant is in intron 1 of the FTO gene (Chr. 16q12.2). Genotyping of rs9939609 and the genetic principal components were performed on whole blood using the TaqMan assay (Applied Biosystems, Foster City, CA, USA), utilizing the Affymetrix 6.0 single nucleotide variant array, and the Birdseed calling algorithm^[37]^ at the Broad Institute Center for Genotyping and Analysis. Allele detection was carried-out using the ABI Prism 7700 Sequence Detection System (Applied Biosystems). We extracted FTO rs9939609 variant data from ARIC genome-wide association study using the software Plink.^[38]^

### 2.4 Statistical analysis

The original participant sample included 14,928 participants at baseline. We imputed missing observations on leisure-time sports physical activity, BMI, and education level to augment our sample, especially for African Americans. Imputations were <2% of the original participant sample. To assimilate an ambulatory population, participants were excluded from the analysis at baseline and follow-up if they had mean arterial pressure <60 mm Hg (n = 117), if there was a difference of <20 mm Hg between systolic and diastolic blood pressures (n = 31), if systolic blood pressure was <80 mm Hg (n = 11), or if diastolic blood pressure was <45 mm Hg (n = 110). Additional participants were excluded if they had missing observations at baseline for MetS component risk factors (n = 315), FTO rs9939609 variant (n = 2179), or the first 3 genetic principal components to control for admixture (n = 1807). Our final models included 10,358 participants, of which 8170 (78.9%) were White and 2188 (21.1%) were African American. The Hardy–Weinberg equilibrium test for rs9939609 was performed using the Chi-Squared goodness-of-fit test for Whites and African Americans separately. Generalized estimating equation regression analyses to estimate population-average risks with risk ratios (RR) and 95% confidence intervals (CI) were used to evaluate the relationship between FTO rs9939609 variant and risk for MetS and its components over time. In these models, we examined the average longitudinal change in MetS and its component risk factors with repeated measures from visit 1 (baseline) to visit 4 (11 years) separately for Whites and African Americans and by rs9939609 variant (i.e., AT moderate-risk carriers or AA high-risk carriers compared to TT low-risk carriers). Among the repeated responses at each visit, we used the exchangeable correlational structure in the main models and the independent correlation structure in stratified and interaction analyses as indicated by Akaike Information Criterion testing. Robust standard errors were used to approximate the variance correctly.^[39]^

All potential risk factors and covariates were tested for inclusion in models. Each MetS component risk factor was defined as 0 = value in the desired healthy range and not medically treated or 1 = risk value for MetS component or medically treated. MetS component risk factors were summed to create the MetS variable. Participants having at least 3 of the 5 MetS component risk factors were identified as having MetS. In order to choose parsimonious models for our study, we used Wald tests in model building. We intentionally did not include covariates for whether participants were current drinkers and current smokers, following testing that demonstrated no significant differences in Wald tests estimates for rs9939609 between the model that included these covariates and a model that did not. Pearson Chi-Squared tests of hypothesis for independence for categorical variables and One-way ANOVA test for continuous variables were applied to examine differences in genotype distributions with risk factors among each racial group separately. Current drinker (yes/no) as presently drinks alcohol weekly, current cigarette smoker (yes/no) as presently smokes cigarettes weekly, type 2 diabetes (yes/no), coronary heart disease (yes/no), and stroke (yes/no) were used to describe the sample (shown in Table 1) but were not used in other analyses.

**Table 1:**
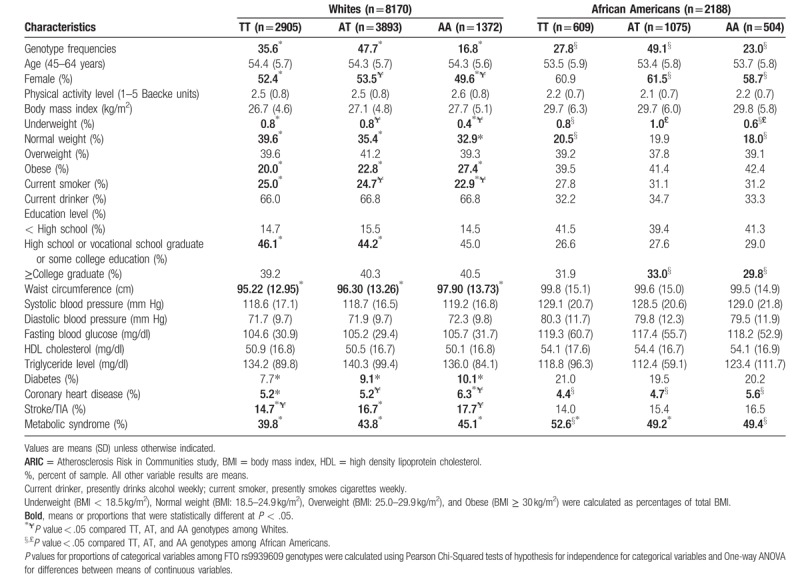
Baseline characteristics of participants by race and FTO rs9939609 genotype: The ARIC study.

Parsimonious models for rs9939609 variant and MetS adjusted for age, gender, physical activity level, education level, duration of study time, and the first 3 genetic principal components to correct for population genetic admixture stratified by Whites or African Americans were chosen as the final models for presentation in the main effects models. In analyses to investigate the probability of either remaining free of MetS over time at each visit, we calculated the time between study visits as the midpoint of each interval (in years) plus the time contributed from the preceding visit.

In stratification models, we used normal BMI as BMI < 25.0 kg/m^2^, and overweight/obese as BMI ≥ 25.0 kg/m^2^. Physical activity (Baecke units, 1–5) was dichotomized at the breakpoint for Whites as ≤1.6 = low physical activity and >1.6 = high physical activity, and for African Americans as ≤2.1 = low physical activity and >2.1 = high physical activity. To test the 3-way interaction of rs9939609 variant, BMI, and physical activity (i.e., rs9939609 × BMI × physical activity) by race, we used available ARIC data as the linear combination of rs9939609, BMI, and/or physical activity, adjusted for covariates. All regression analyses were bootstrap with 10,000 repetitions. Statistical analyses were conducted using Stata MP, version 15.0 (StataCorp, College Station, TX).

## 3 Results

### 3.1 Descriptive characteristics

At baseline (1987–1989), there were 8,170 Whites (3,479; 42.6%) and 2188 African Americans (1,098; 50.2%) with MetS aged 45 to 64 years. The number (percent) of participants at baseline who met the old MetS criteria that included blood pressure cut-points of ≥130 mm Hg systolic and ≥85 mm Hg diastolic was 3194 (37.1%) Whites and 1042 (47.67%) African Americans. Using these older blood pressure criteria (i.e., ≥130/≥85 mm Hg), the prevalence of MetS during the 11-year follow-up period was 46.4% in Whites and 54.2% in African Americans. In contrast, using the updated criteria for elevated blood pressure (i.e., ≥120/≥80 mm Hg), the prevalence of MetS was 50.3% in Whites and 57.0% in African Americans. The FTO rs9939609 variant was found to be in Hardy–Weinberg equilibrium (*P* > .05) in both racial groups.

Table 1 shows baseline characteristics of participants in our study stratified by race and rs9939609 genotypes (TT = low risk, AT = moderate risk, and AA = high risk). Genotype frequencies were statistically different within both racial groups. In general, the proportion of participants with normal BMI, BMI ≥ 30 kg/m^2^, females, current smokers, high waist circumference, type 2 diabetes, coronary heart disease, stroke/TIA, or MetS was significantly different between rs9939609 genotypes among Whites. However, only coronary heart disease and MetS were significantly different between rs9939609 genotypes among African Americans.

**Figure 1:**
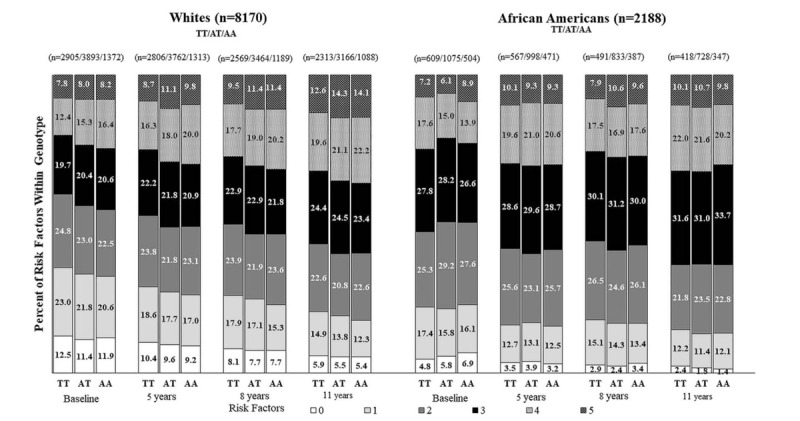
Percent of participants within TT, AT, and AA genotypes at baseline and over time with MetS component risk factors (i.e., high waist circumference, high blood pressure, high fasting blood glucose, low HDL (high density lipoprotein) cholesterol, and high triglycerides) in the ARIC study.

Figure 1 displays the 4 visits by number of MetS component risk factors (0, 1, 2, 3, 4, and 5) by race and rs9939609 genotypes. African Americans accumulated a higher percent of ≥3 MetS component risk factors than Whites across visits, but percentages among Whites were generally higher in the TT low-risk carriers compare to AT and AA higher-risk carriers. More specifically, across all visits from baseline to 11 years of follow-up, a lower percentage of African American rs9939609 TT, AT, and AA carriers had 0, 1, and 2 MetS component risk factors and a higher percent had 3 and 4 MetS component risk factors. However, a smaller proportion of African Americans had 5 MetS component risk factors than Whites. As MetS component risk factors increased over time, the percent of participants with 0, 1, and 2 MetS component risk factors became progressively smaller across all visits, moreso in African Americans than Whites. The prevalence of 3, 4, and 5 MetS component risk factors (defined as having MetS) increased in both Whites and African Americans across visits, with more African Americans having 3 or more MetS component risk factors than Whites.

**Figure 2:**
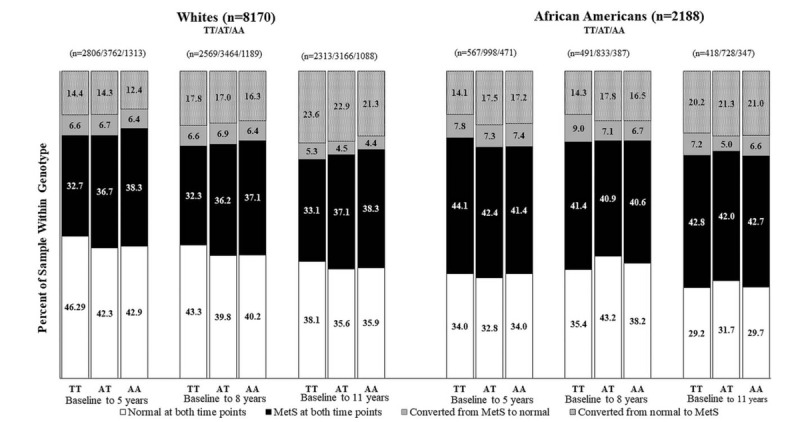
Percentage of participants within TT, AT, and AA genotypes at baseline (visit 1) and through 11 years of follow-up (visit 4) who remained free of metabolic syndrome (≤2 risk factors), those who remained with metabolic syndrome (≥3 risk factors), those who converted from having metabolic syndrome to not having metabolic syndrome (i.e., decreased from ≥3 risk factors to ≤2 risk factors), and those with converted from without metabolic syndrome to having metabolic syndrome (i.e., increased from ≤2 risk factors to ≥3 risk factors) in the ARIC study. X-axis description for time in study: Baseline (visit 1); 5 years (end of visit 2); 8 years (end of visit 3); 11 years (end of visit 4).

Figure 2 shows the proportion of participants with MetS and without MetS and those who reversed from MetS to without MetS. Across all visits, White AT and AA higher-risk carriers generally had a higher proportion of MetS than TT low-risk carriers over time. Among participants without MetS, a lower percentage of Whites were AT and AA higher-risk carriers compared to TT low-risk carriers, while for African Americans, there is no clear pattern. African Americans who converted from having MetS to not having MetS were more likely to be AT and AA higher-risk carriers than TT low-risk carriers.

**Figure 3:**
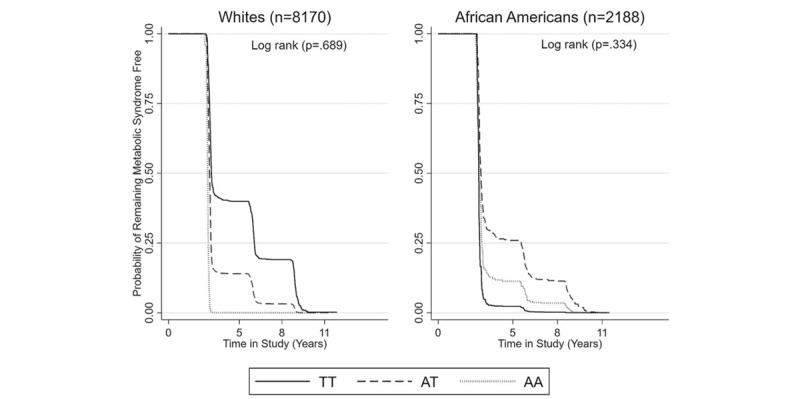
Estimated probability by race for those who remained free of metabolic syndrome (≤2 risk factors) at each visit. Log rank test showed no significances differences in survival functions between FTO rs9939609 (TT, AT, and AA carriers) for Whites (*P* = .686) and African Americans (*P* = .334). Time in study (Years): 0 = baseline (visit 1), 5 = end of visit 2, 8 = end of visit 3, 11 = end of visit 4.

Figure 3 displays the longitudinal changes in metabolic syndrome across time, by race, for those who remained free of MetS and first conversion to MetS at each visit. At time zero, all participants were free of MetS. However, with time, participants converted to MetS such that at about 3 years, all White AA high-risk carriers converted to MetS; 86% of AT moderate-risk carriers and 55% of TT low-risk carries converted to MetS. Among African Americans at about 3 years into the study, nearly all TT low-risk carriers converted to MetS; 65% of AT and 86% of AA higher-risk carriers converted to MetS. There was no clear relationship with FTO rs9939609 genotypes among African Americans, unlike among Whites. In Figure 3A and 3B, the log rank test for equality in survival was not significantly different between genotypes by race.

### 3.2 FTO rs9939609 polymorphism and MetS component risk factors

Table 2 shows the 5 components of MetS. Among the 5 MetS components, FTO rs9939609 variant was associated with the highest magnitude of risk for higher waist circumference in Whites but not in African Americans. In general, White participants with elevated waist circumference who had 2 high-risk alleles (AA carriers) had a higher risk (Risk Ratio (RR) = 1.12; 95% confidence interval: 1.10–1.14) than those with 1 high-risk allele (AT-allele carriers; RR = 1.07; 1.06–1.09). Whites who carry the A-allele (AT and AA genotypes) had 6% higher risk (RR = 1.06; 1.05–1.07) for elevated waist circumference. Likewise, we observed small significant associations with elevated blood pressure, high fasting blood glucose, low HDL cholesterol and high triglyceride levels for AA and AT higher-risk carriers compared to TT low-risk carriers among Whites (*P* < .05). These main-effects relationships were observed only for high triglyceride levels among African Americans AA high-risk carriers over time (RR = 1.11; 1.02–1.21)

**Table 2:**
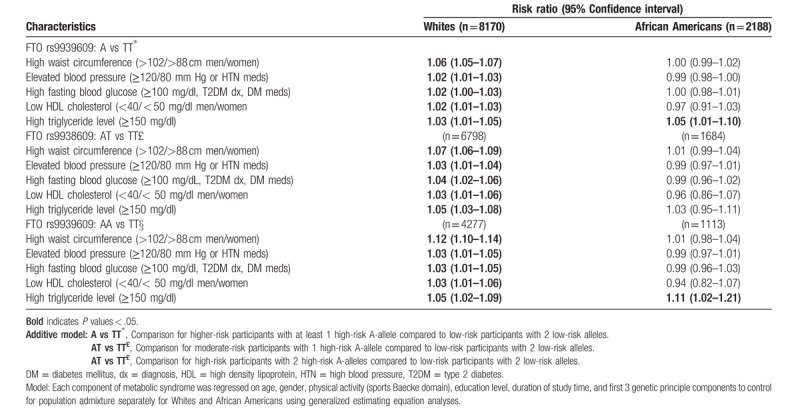
FTO rs9939609 and increase risk in metabolic syndrome component risk factors by race over time in the ARIC study.

### 3.3 FTO rs9939609 variant and MetS

Among Whites in main effects models, the FTO rs9939609 variant was significantly associated with small increases in risk for MetS over time after adjusting for covariates (Table 3). Whites with 1 high-risk allele (AT moderate-risk carriers) and 2 high-risk alleles (AA high-risk carriers) had 1.07 and 1.08-fold increase in the risk for MetS (*P* < .05) over 11 years of follow-up. Among Whites, BMI nullified the effect estimates of FTO rs9939609 on MetS and therefore was not included in the models in Table 3. Statistically significant effects were not seen among African Americans.

**Table 3:**
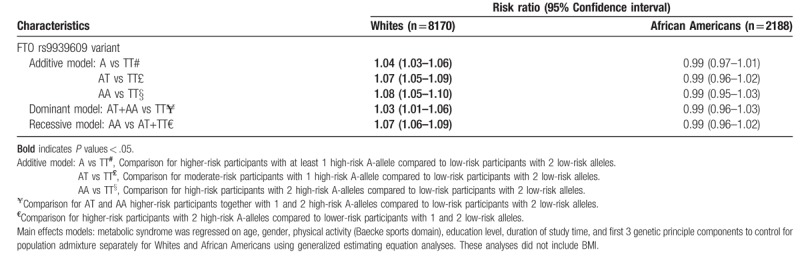
FTO rs9939609 and longitudinal changes in metabolic syndrome by race over time in the ARIC study.

One of the underlying reasons for assessing dominant and recessive modes of inheritance is to see whether participants with at least 1 high-risk allele (dominant mode) or 2 high-risk alleles (recessive mode) have an increased risk for MetS. We used the method of the QIC to select the best fitting model. Because generalized estimating equation is based on a correlation structure, the correlation matrix had the smallest Akaike Information Criterion (QIC) value across the different equations in the main models. In stratified models with BMI and physical activity, the independent correlation structure had the lowest QIC among different correlational structures and this was used as the preferred correlational structure in stratified models. In main models, we compared the additive A-allele, dominant and recessive equations, for Whites and African Americans separately. For Whites, the values were: additive A-allele mode QIC = 7109.193, dominant mode QIC = 7158.258, and recessive mode QIC = 7118.232. For African Americans, the corresponding values were: additive A-allele mode QIC = 6115.160, dominant mode QIC = 6124.590, and recessive mode QIC = 6116.776. Our results suggest that the additive A-allele QIC was the smallest, but differences within races were small.

### 3.4 FTO rs9930609, BMI, and physical activity in relation to MetS

Table 4 shows the effects of high and low levels of BMI and physical activity on MetS by race. Among Whites, we observed mostly small effects for FTO rs9939609 on MetS for overweight/obese participants. Within strata of high and low physical activity, participants with low physical activity level had higher risks for MetS than participants who reported high physical activity. The risk was higher for AT carriers, but the confidence intervals were very wide, indicating imprecision of the estimate, most likely due to small sample size. Most importantly, only the overweight/obese category and low and high physical activity categories among Whites met Bonferroni correction for multiple testing. Among African Americans, there were no statistically significant effects for high or low physical activity or for overweight or obese categories. Only the FTO rs9939609 × BMI interaction in Whites was statistically significant.

**Table 4:**
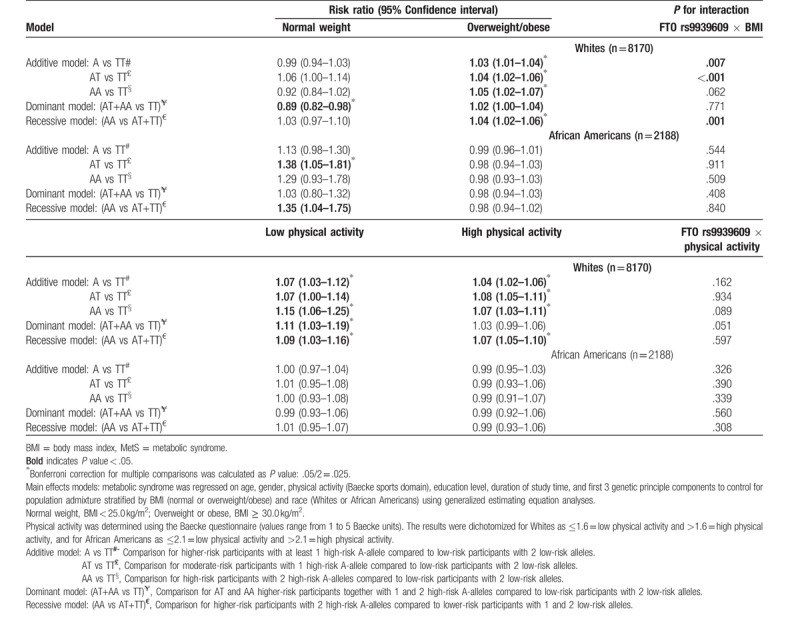
Associations between FTO rs9930609 and MetS over time stratified by BMI or physical activity and race in the ARIC study.

**Figure 4:**
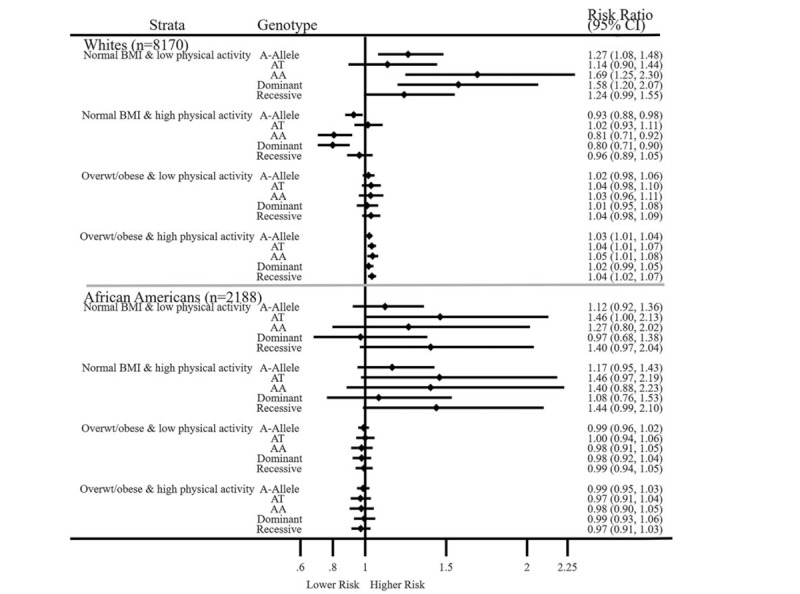
Associations of FTO rs9930609, BMI, and Physical Activity in Relation to MetS. overwt, overweight. Normal weight, BMI < 25.0 kg/m^2^; Overweight or obese, BMI ≥ 25.0 kg/m^2^. Physical activity was determined using the Baecke questionnaire (values range from 1 to 5 Baecke units). Physical activity was dichotomized for Whites as ≤1.6 = low physical activity and >1.6 = high physical activity, and for African Americans as ≤2.1 = low physical activity and >2.1 = high physical activity. ^∗^Bonferroni correction for multiple comparisons was calculated as *P* value: .05/2 = .025. Metabolic syndrome was regressed on age, gender, physical activity (Baecke sports domain), education level, duration of study time, and the first 3 genetic principle components to control for population admixture separately for Whites and African Americans using generalized estimating equation analysis. All models were stratified by combinations of high or low BMI or high or low physical activity. The *P* values for interactions for FTO rs9939609 × BMI × physical activity were as follow, Whites: A-allele *P* < .001, AT *P* = .558, AA *P* = .140, dominant *P* = .002, and recessive *P* = .747; African Americans: A-allele *P* = .028, AT *P* = .060, AA = .010, dominant *P* = .039, and recessive *P* = .013.

Figure 4 shows the combinations of stratifications of FTO rs9939609, low and high BMI, and low and high physical activity on MetS. Among Whites with normal BMI and low physical activity, higher risks for MetS were seen among FTO rs9939609 higher-risk carriers. However, a high physical activity level was protective in participants with normal BMI. Among Whites reporting a high physical activity level who were overweight or obese, slightly higher risks were seen. Among African Americans, most risks were not significant, except that AT moderate-risk carriers with normal BMI and low physical activity had a 1.46-fold increased risk for MetS (RR = 1.46; 1.00–2.13). However, the confidence interval was wide, indicating imprecision, and the lower end bordering non-significance.

Figure 5 A and 5B show the effect estimates of the variables and their interactions adjusted for covariates and other interaction terms in relation to MetS. The highest risks were seen among Whites for the effect of FTO rs9939609 alone in higher-risk carriers. In both racial groups, each additional BMI unit was associated with small increases in risk for MetS. However, physical activity alone was independently protective of MetS in FTO rs9939609. Among Whites, the protective effects of physical activity on MetS remained significant even after Bonferroni correction for multiple testing when we compared risks for FTO rs9939609 × BMI vs FTO rs9939609 × BMI × physical activity in AT moderate-risk carriers. Statistically significant effects were less common among African Americans.

**Figure 5:**
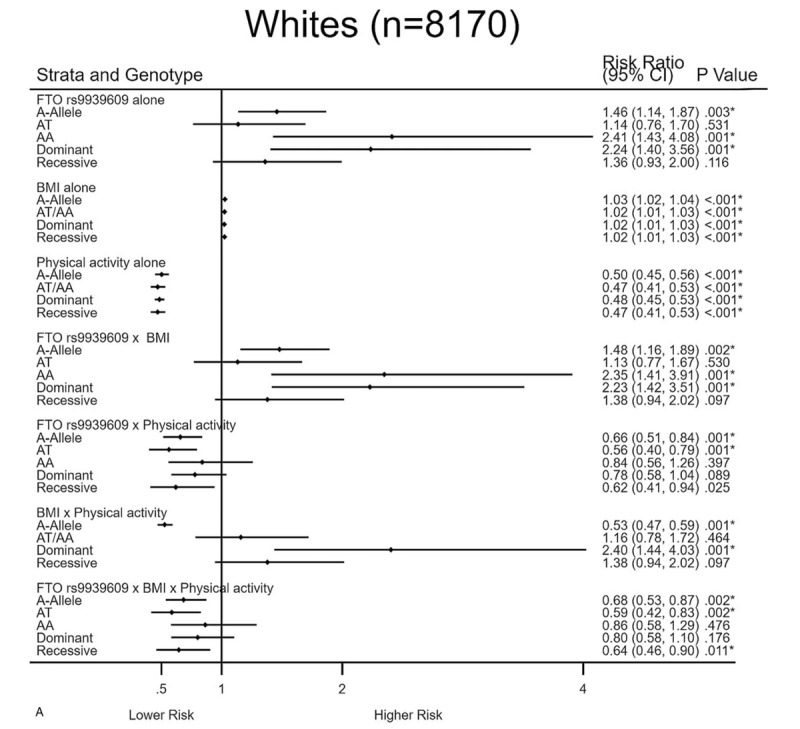
A and B. Interactions between FTO rs9939609 × BMI × Physical activity in relation to MetS. Interaction models: Metabolic syndrome was regressed on FTO rs9939609, age, gender, physical activity (Baecke sports domain), body mass index, education level, duration of study time, the first 3 genetic principle components to control for population admixture, and interactions for FTO rs9939609 × BMI × physical activity, for Whites and African Americans separately using generalized estimating equation model. ^∗^Bonferroni correction for multiple comparisons was calculated as *P* value: .05/4 = .013.

**Figure 5 (Continued):**
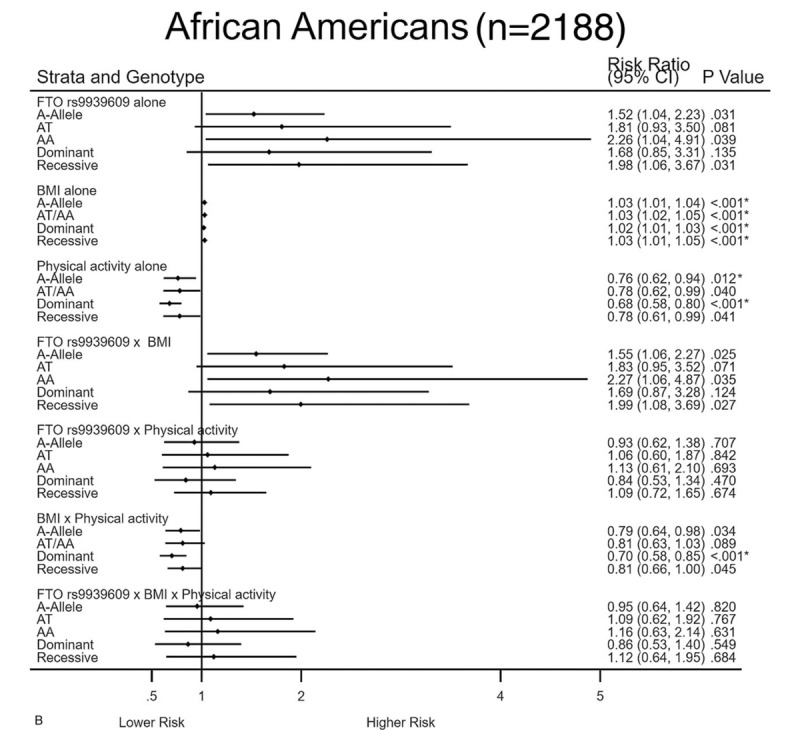
A and B. Interactions between FTO rs9939609 × BMI × Physical activity in relation to MetS. Interaction models: Metabolic syndrome was regressed on FTO rs9939609, age, gender, physical activity (Baecke sports domain), body mass index, education level, duration of study time, the first 3 genetic principle components to control for population admixture, and interactions for FTO rs9939609 × BMI × physical activity, for Whites and African Americans separately using generalized estimating equation model. ^∗^Bonferroni correction for multiple comparisons was calculated as *P* value: .05/4 = .013.

## 4 Discussion

We observed that the prevalence of MetS in the ARIC population was 50.3% in Whites and 57% in African Americans using the updated criteria for elevated blood pressure (≥120/≥80 mm Hg), compared to 46.4% in Whites and 54.2% in African Americans when using the older criteria (≥130/≥85 mm Hg). Among MetS component risk factors, waist circumference was the most prevalent among Whites with AT and AA genotypes. FTO rs9939609 variant was significantly associated with high triglyceride level in African Americans. Our findings show that FTO rs9939609 variant had small contributions to increased risk for MetS in Whites in the main effects models. Among Whites, high levels of physical activity were protective against deleterious effect of FTO rs9939609 on MetS, regardless of whether they had normal BMI or were overweight or obese.

In other studies, de Luis et al^[15]^ found that females with the rs9939609 TT low-risk variant without MetS but who were obese had higher levels of insulin resistance, insulin, and triglycerides using the old MetS criteria with higher cut-points for blood pressure. It is likely, however, that some participants in that study who had <3 MetS risk factors would be categorized as having MetS using the updated criteria for elevated blood pressure.

Al-Attar et al^[19]^ found that the FTO rs9939609 variant was associated with MetS in a non-White population, a finding that was not observed in our study, possibly due to relatively small sample size of African Americans. The prevalence of MetS is known to increase with rising BMI. Similar to the Korean study by Biak et al^[31]^ we found that BMI modified the effect of the FTO rs9939609 variant on MetS over time in Whites. The interactions of rs9939609 variant with BMI increased the risk ratios above that of the main effects models among Whites. However, among Whites, the risk ratios became protective when physical activity was included in the interaction model (rs9939609 × BMI × physical activity). Oyeyemi et al found that FTO rs9939609 × physical activity interaction lowered the risk of overweight/obesity and increasing BMI in a Nigerian population.^[40]^

Rampersaud et al^[23]^ found that physical activity blunted the effects of obesity in an Amish population. In another ARIC study analysis, Demerath et al^[24]^ found an interaction with FTO rs9939609 variant and physical activity on BMI, waist circumference, and skinfold thickness among White and African American men. Moreover, Demerath et al^[24]^ and Andreasen et al^[22]^ found that high physical activity levels decreased the effect of rs9939609 variant on body fat accumulation and adiposity in their cross-sectional studies. Like Demerath et al^[24]^ we also observed interactions with rs9939609 variant, BMI and physical activity on risk of MetS over time in the ARIC sample, but among Whites only. Furthermore, the overall effect of high levels of physical activity had protective independent effects on MetS in both racial groups (*P* < .05). The risks for MetS were highest among Whites for the independent effect of FTO rs9939609 in higher-risk carriers. Each additional unit of BMI had small increases in risk for MetS, such that a BMI of 25 and 30 kg/m^2^ would convey relative risks of 1.79 (1.40–2.28) and 2.01 (1.50–2.69), respectively. However, higher levels of physical activity had independent protective effects against MetS in both racial groups (*P* < .05) and blunted the adverse effects of higher BMI.

An important aspect of our study was that we used an updated definition of the MetS, based on the current blood pressure guidelines, which were revised in 2017.^[5]^ Use of the current blood pressure guidelines (vs the older criteria) increased the prevalence of MetS in Whites and African Americans from 41.4% to 49.4% at baseline and from 49.2% to 56.8% at the follow-up time points.

We identified a few limitations in our study. The sample of African Americans in the ARIC cohort was smaller than for Whites, particularly after excluding participants with missing values for covariates. This may have contributed to non-significant findings in our results. African Americans are reported to have lower rates of MetS than Whites.^[41]^ Despite finding higher rates for MetS among African Americans than Whites in our sample, we did not observe any significant associations of rs9939609 with MetS in the main effects models. Some reports claim that the lack of association of FTO rs9939609 variant and MetS among African Americans may be due to the differences in linkage disequilibrium patterns and that this genetic effect may be captured by different markers in Whites than African Americans.^[42]^ However, in our analysis, through interactions with BMI and physical activity, we observed higher risks that were statistically significant for AT moderate risk carriers among African Americans, but confidence intervals were wide, which demonstrated low statistical power or a chance finding. Our analyses may also contain residual confounding due to the exclusion of dietary factors. Nonetheless, the effects we found among Whites and African Americans in our longitudinal study extended the results of cross-sectional studies.

In summary, these findings suggest that the interaction of FTO rs9939609 variant and BMI can significantly increase the risk for MetS among Whites over time and that physical activity can decrease these risks. Our findings also indicate that the association of the FTO rs9939609 variant with MetS may be ancestry-specific, which is supported by the lack of significance among African Americans in the ARIC sample. In addition, the interactions of rs9939609 variant with BMI and physical activity on MetS risk differed based on ancestry. The longitudinal aspect of this study provides evidence for a genetic effect of the FTO rs9939609 variant among people of European ancestry (Whites) on risks for MetS, even after accounting for age, gender, physical activity, education level, duration of follow-up time, and population admixture genetic biases. The observation that physical activity may attenuate the effect of the rs9939609 variant and higher BMI on MetS risk adds to the well-documented benefits of physical activity and supports the recommendations of the Physical Activity Guidelines for Americans.^[43]^ It will be important to replicate these genetic analyses in future studies with a larger sample of African Americans.

## Author contributions

**Conceptualization:** Dale S Hardy.

**Formal analysis:** Dale S Hardy, Susan B Racette.

**Funding acquisition:** Dale S Hardy, Tesfaye B Mersha.

**Investigation:** Dale S Hardy.

**Methodology:** Dale S Hardy, Jane T Garvin, Tesfaye B. Mersha, Susan B Racette.

**Project administration:** Susan B Racette.

**Supervision:** Dale S Hardy, Susan B Racette, Tesfaye B. Mersha.

**Visualization:** Jane T Garvin.

**Writing – original draft:** Dale S Hardy, Jane T Garvin, Susan B Racette.

**Writing – review & editing:** Dale S Hardy, Jane T Garvin, Tesfaye B Mersha, Susan B Racette.
